# The Intersection of Personality Dimensions with Stress Relief Strategies in Adolescence: An Experimental Study

**DOI:** 10.3390/ijerph21121650

**Published:** 2024-12-10

**Authors:** Jeroen Pronk, Iris Eekhout, Katharina Preuhs, Olmo van der Mast, Renate van Zoonen, Symone B. Detmar

**Affiliations:** Expertise Group Child Health, the Netherlands Organization for Applied Scientific Research (TNO), P.O. Box 2215, 2301CE Leiden, The Netherlands; iris.eekhout@tno.nl (I.E.); katharina.preuhs@tno.nl (K.P.); olmo.vandermast@tno.nl (O.v.d.M.); renate.vanzoonen@tno.nl (R.v.Z.); symone.detmar@tno.nl (S.B.D.)

**Keywords:** stress, adolescence, physical exercise, mindfulness, personality, personalized feedback

## Abstract

Stress is becoming more prevalent among adolescents and negatively impacts their health and development. It is, therefore, pivotal to increase our knowledge about potential (personalized) healthy stress relief strategies for adolescents. This study investigated individual personality differences (i.e., behavioral inhibition versus behavioral activation) in adolescents’ preference for, and the effectiveness of, physical or mental exercise to relieve stress. A sample of 208 adolescents (12 to 18 years) were recruited during a science and education student festival in the Netherlands. For ethical reasons, no personally identifiable information could be collected. Surveys were used to assess personality and preferences at baseline and subjective stress at baseline, after stress induction with the Sing-a-Song Stress Test, and after stress relief through physical or mental exercise. The results from multivariate regression analyses indicate that personality did not significantly influence adolescents’ preference for, or benefit from, physical or mental exercise for stress relief. Both types of exercise significantly reduced experienced stress, but the effect was stronger when adolescents performed their activity of choice. The findings suggest that pre- and intervention efforts for adolescents’ stress-related health problems are better directed at offering a range of effective free-choice stress relief activities than on personalized stress-relief methods.

## 1. Introduction

Stress has been classified as a primary and growing health concern among adolescents [1,2,3]. The systematic review indicates a significant increase in adolescent stress in recent years and that currently, one in four to five adolescents suffers from stress-related health issues [4,5]. This increase in stress among adolescents has been attributed to recent social transformations in society, such as digitalization (e.g., social comparison and bullying through the internet and social media), increased individualism (e.g., increased academic and physical performance pressures), and changes in family dynamics (e.g., parental conflict, economic instability [3,6]). Stress is generally defined as the mental and/or physical tension that is experienced in response to challenging situations [7]. These challenging situations can be acute events (e.g., speaking in public) or more chronic circumstances (e.g., peer relationship difficulties). Acute stress can be a natural and (generally) evolutionary adaptive response that activates the body via the nervous system to take action in response to a stressor [8]. Chronic stress and ineffective coping with stress, however, can have detrimental effects on adolescents’ academic performance [9], as well as on their overall physical (e.g., headaches, cardiovascular issues, immune system suppression), mental (e.g., anxiety, depression, cognitive impairments), and social (e.g., aggression, social isolation, relationship strain) health and well-being [2,6,10,11]. Moreover, from a developmental perspective, adolescents are more likely to engage in risky behaviors, resulting in more unhealthy stress relief strategies (e.g., substance abuse, delinquency, self-harm, e.g., [12]). While often effective in reducing stress in the short term, these strategies may cause and/or exacerbate physical, mental, and social health and well-being issues in the medium and long term. Due to the increasing prevalence of stress and stress-related health problems among adolescents, it is pivotal to increase our knowledge of effective and healthy stress relief strategies that adolescents may incorporate into their daily lives.

Stress relief entails the release of experienced mental or physical tension. Various structural and healthy stress relief strategies have been recommended by the World Health Organization [13]. These include developing a daily routine, maintaining a healthy diet, and getting enough sleep. For more immediate stress relief, it is recommended to engage in physical exercise (e.g., running) and/or mental exercise (e.g., mindfulness [2,14,15]). Besides relieving stress and improving adolescents’ overall health and well-being [2,13], both of these strategies are also cost-efficient. However, it is likely that there are individual differences in the effectiveness of and in adolescents’ preference for either of these strategies for stress relief. We hypothesize that individual personality differences are a plausible candidate in this regard. The present study, therefore, investigated whether the preference for and effectiveness of stress relief strategies for acute stress relief are influenced by individual differences in personality.

Personality refers to the complex pattern of behavioral, cognitive, and emotional characteristics an individual exhibits in their daily lives [16]. Particularly relevant in explaining individual differences in stress and stress response is the Reinforcement Sensitivity Model (RSM; [17,18]). Grounded within neurobiological mechanisms and theory, the RSM explains individual differences in personality through two interconnected regulatory systems [17,18,19]: (1) the behavioral inhibition system (BIS), which is related to inhibitory processes, withdrawal from stimuli, sensitivity to punishment, and negative affect; and (2) the behavioral activation system (BAS) which is related to excitatory processes, approach towards stimuli, sensitivity to reward, and positive affect.

The BIS has been found to predispose individuals to stress and is associated with higher stress vulnerability [20,21,22]. BIS-sensitive youth and adults were also found to manage stress better by withdrawing from stressors through mental exercise [22,23]. In contrast, the BAS has been related to heart rate reactivity and the use of active coping strategies to manage stress [21]. BAS-sensitive adolescents were also found to benefit more from active stress relief strategies to manage their stress [24]. Following from this, we hypothesize that (1) adolescents with a stronger BIS profile will exhibit a stronger stress relief response to mental exercise as this focuses on withdrawing from external stimuli and circumstances, while (2) adolescents with a stronger BAS profile will exhibit a stronger stress relief response to physical exercise as this focuses on coping with stress through a physically rewarding and energizing activity. Furthermore, we also explored whether (1) BIS/BAS personality differences also impact adolescents’ preference for mental or physical exercise, respectively, and (2) adolescents who engage in their preferred stress relief activity have a stronger stress relief response.

In sum, stress prevalence is increasing among adolescents and can negatively impact their development, health, and well-being. It is, therefore, pivotal to teach adolescents effective and healthy strategies that can help them to relieve experienced stress. While such strategies, such as engaging in physical or mental exercise, are available, it is currently still unclear whether their effectiveness is influenced by individual differences in strategy preference and stress experience. Thus, the strategy effectiveness of physical and mental exercise for stress relief may be susceptible to individual differences. The present experimental study was designed to overcome this knowledge gap by investigating whether these individual differences could be explained by BIS/BAS personality differences in a sample of Dutch adolescents. We predicted that adolescents with a stronger BIS profile would benefit more from mental exercise (i.e., mindfulness) than from physical exercise (i.e., trampoline jumping) to relieve experimentally induced social stress. Conversely, we predicted that adolescents with a stronger BAS profile would benefit more from physical than from mental exercise to relieve experimentally induced social stress.

## 2. Materials and Methods

### 2.1. Participants

Data (i.e., excluding personally identifiable data such as age and gender) were collected at a science and education festival for secondary school students in the central part of the Netherlands in June of 2023. All students visiting the festival could choose to participate in the study. Parents of students younger than 16 years were sent a passive informed consent letter via the festival organization before the festival and could object to their child’s participation in the experiment by returning a preprinted objection note. Students themselves provided their active, informed consent by indicating whether they wanted to participate in the study at the start of the testing procedure and could cease participation at any time during testing. This study and the procedures used were approved by the authors’ institutional review board (IRB; #2023-052). A total of 208 secondary school students (aged 12 to 18 years) participated in this study at baseline (i.e., T1). Not all participants also provided data on all three time points (i.e., after stress induction, or T2: *N* = 204, and after stress relief, or T3: *N* = 188). However, there were no differences in any of the T1 study measures between participants who provided data at all time points and those who did not (i.e., subjective stress experience at T1, BIS, BAS, and activity preference; all: *p* ≥ 0.40).

### 2.2. Materials and Measures

#### 2.2.1. Subjective Stress Experience

Participants’ subjective stress was measured with the item “What is your current stress level?” on a scale ranging from 1 (“not at all”) to 10 (“extremely”). Subjective stress was measured at T1 (M = 4.11, SD = 2.20) and after both experimental activities (T2: M = 4.61, SD = 2.58; T3: M = 3.42, SD = 2.31).

#### 2.2.2. Personality

Participants’ personality conforming to BIS and BAS was measured with the constructs of sensitivity to punishment (for BIS) and sensitivity to reward (for BAS) of the Dutch 10-item Sensitivity to Punishment and Sensitivity to Reward Questionnaire (SPSRQ-D10; [25]). The SPSRQ-D10 is based on the original 48-item Sensitivity to Punishment and Sensitivity to Reward Questionnaire [26]. See Pronk and colleagues [25] for a full description of the SPSRQ-D10, as well as reliability and validity information. With regards to construct validity specifically, BIS and BAS, as assessed through the SPSRQ-D10, were associated with the Big Five personality domains and bullying-related outsider- and defender-behavior as expected based on theory and expectation [25]. BIS (example item: “I am a shy person”) and BAS (example item: “I often do things to be praised”) were measured with 5 items each on a scale without a neutral response option ranging from 1 (very untrue) to 4 (very true). The final variables were calculated as mean scores with Cronbach’s alpha reliability coefficients of 0.73 for BIS (M = 2.13; SD = 0.64) and 0.64 for BAS (M = 2.13; SD = 0.61).

#### 2.2.3. Stress Inducing Activity

Experimental stress was induced using a short adapted version of the Sing-a-Song Stress Test (SSST; [27]). The SSST version used in this study consisted of three task rounds of 1 min each. Participants were sat in a semi-circle facing each other while being confronted with a visual timer counting back from 60 to 0 s. Participants were instructed not to share or show their tasks to other participants or talk with each other. In the first round, participants received an individual silent cognitive task (e.g., mentally listing countries starting with the letter D). In the second round, participants received a simple physical action task (e.g., crossing their arms when the timer reached 0). Both of these tasks, and the second task specifically, served the purpose of making participants believe all tasks were individualized. Finally, in the last round, social stress was induced in participants by instructing them to sing their favorite song when the timer reached 0. The SSST has been proven effective in inducing physiological stress in adult samples [27,28].

#### 2.2.4. Stress Relief Activity

For stress relief, participants were randomly assigned to one of two 3 min individual activities: (1) trampoline jumping while listening to music on a headset (i.e., physical exercise) or (2) guided breathing mindfulness exercise via a headset in a relaxing pose (i.e., mental exercise).

### 2.3. Procedure

An experimental round lasted for approximately 10 min. A maximum of eight participants could participate in each round. At the start of an experimental round, participants received a short study introduction (i.e., participation in a study about stress relief, that is, the release of experienced tension), provided their digital consent, and filled in the first part of the questionnaire (i.e., the SPSRQ-D10, baseline subjective stress (T1), and preferred stress relief activity). Participants were then randomized into one of the stress relief activities using a random number generator and asked to participate in a game, the SSST (i.e., stress-inducing activity). The rules of the SSST were explained without revealing the nature of the activity. Following the SSST, participants were asked to report their subjective stress after stress induction (T2) and assigned to their individual 3 min stress relief activity (i.e., physical or mental exercise). Finally, after the stress relief activity, participants provided their subjective stress experience after stress relief (T3). After completing the experimental procedure, participants received personalized feedback relating to their (1) personality profile, (2) (change in) stress level, and (3) stress relief activity (including [mis]match with the hypothesized most effective stress relief strategy). Participants could email themselves this feedback (personal data were not stored).

## 3. Results

### 3.1. Preliminary Analyses

Four types of preliminary analyses were executed. First, the frequency distributions for both stress relief activities were analyzed with Chi-square tests to assess whether participants’ activity preferences matched with the activity performed. The contingency table for these analyses is presented in Table 1. Participants were equally divided into the two stress-relieving activities (X^2^ [1, 208] = 0.08, *p* = 0.781) but were not equally divided in preference for a stress-relieving activity (X^2^ [1, 207] = 25.74, *p* < 0.001). The majority of participants preferred the physical exercise (i.e., 67.3%), of which about 50% were also assigned to perform the physical exercise.

Second, the associations between activity preference and personality (i.e., BIS and BAS) were analyzed with Welch Two Sample *t*-tests. Students with a preference for mental exercise (M = 2.27; SD = 0.66) had a significantly higher BIS score than students with a preference for physical exercise (M = 2.02; SD = 0.60), t(122.83) = 2.01, *p* = 0.046, d = 0.30. There were no significant differences between students with a preference for mental exercise (M = 2.18; SD = 0.62) and those with a preference for physical exercise (M = 2.07; SD = 0.62) in terms of BAS scores, t(133.62) = −1.78, *p* = 0.077, d = 0.26).

Third, the correlations between all study variables were calculated (i.e., Pearson coefficients for continuous variable pairs, point-biserial coefficients for combined continuous and categorical variable pairs, and the Phi coefficient for the categorical variable pair) and are presented in Table 2. Subjective stress was positively correlated across time points. Subjective stress was also positively correlated with BIS at all time points. Moreover, activity preference was negatively correlated with both stress at T3 and BIS, indicating that adolescents with a preference for mental exercise reported more stress at T3 and higher BIS scores. No other correlations were significant.

Finally, the average change in subjective stress experience following stress induction and stress relief was analyzed through linear regression analyses. Supporting the construct validity of our subjective stress measure, participants’ stress increased significantly following the stress-inducing activity (i.e., from T1 to T2; b = 0.51, t(188) = 2.79, *p* = 0.011, d = 0.20) and decreased significantly following the stress relief activity (i.e., from T2 to T3; b = −1.26, t(188) = −6.05, *p* < 0.001, d = 0.44).

### 3.2. Main Analyses: Predicting Stress Relief

A stepwise multivariate linear regression model was used to statistically predict stress relief by activity performed, personality (i.e., BIS and BAS), and activity preference. Stress relief was calculated as the decrease in subjective stress from T2 to T3 (i.e., stress relief = subjective stress T2—subjective stress T3). The model and outcomes are presented in Table 3. First, mean-centered subjective stress at T1 and T2 was added into the model to avoid regression to the mean, as well as the main effect of the activity performed. Model 1 shows a stress relief effect from T2 to T3 (i.e., a positive constant). Also, more stress at T1 resulted in less stress relief, and more stress at T2 resulted in more stress relief. Activity performed (i.e., mental or physical exercise) did not significantly predict stress relief. Second, the main effects of BIS and BAS were added to the model. Model 2 shows a significant negative effect of BIS on stress relief, while BAS did not significantly predict stress relief. Third, the interactions between the activity performed and both BIS and BAS were added to the model (each interaction separately). Model 3A shows that these interactions did not significantly impact stress relief. Finally, the interaction between activity preferred and performed was introduced into the main effects model (i.e., Model 2) to gauge the impact of activity preference on adolescents’ stress relief. Model 3B shows that this interaction significantly predicted stress relief. Stress relief was stronger when the activity preferred and performed were aligned.

## 4. Discussion

Stress can have maladaptive developmental and health consequences for adolescents. The present study investigated whether both the effectiveness and preference for specific healthy stress relief strategies are influenced by individual differences in adolescents’ personalities. We expected that adolescents with a stronger BIS profile would benefit more from mental exercise, while adolescents with a stronger BAS profile would benefit more from physical exercise to relieve experimentally induced social stress. While we could not confirm these expectations, we did find that both stress relief activities effectively reduced adolescents’ experienced stress. What is more, we found that the stress relief effects of these activities were stronger when adolescents performed their preferred activity. As such, the findings seem to suggest that adolescents’ stress relief may be best supported by focusing our efforts on offering them a range of healthy stress relief activities to choose from rather than offering just one personalized method.

Counter to expectation and previous work by Heponiemi and colleagues [21] linking BAS sensitivity to active coping with stress, the effectiveness of and preference for physical exercise were not influenced by adolescents’ personality profiles. On the other hand, and consistent with expectations and previous work [20,21], we did find BIS-sensitive adolescents to be more prone to stress and to prefer mental exercise for stress relief. However, the assumed link between BIS sensitivity and increased benefit from stress relief through mental exercise could not be confirmed.

The findings also contradict Jellesma and Cornelis [22], who found that early adolescents with a BIS profile uniquely benefited from yoga-based mindfulness to relieve stress. Differences in findings may be influenced—among other things—by stress measures used. Jellesma and Cornelis [22] used questionnaire measures for stress that strongly tapped into the BIS construct and emphasized emotional and somatic stress complaints. In the present study, stress was manipulated experimentally with the SSST [27]. Moreover, Jellesma and Cornelis [22] exposed a younger adolescent sample to a multi-week stress relief program with weekly multi-activity sessions of 50 min. However, the intensity of the stress relief activities seems an unlikely explanation for differences between studies. In our study, the short 3 min session of (only) mindfulness also resulted in significant stress reductions in adolescents.

Finally, the SSST, which we used to experimentally induce stress, has—to our knowledge—only been used in adult samples until now with evidence of physiological stress induction [27,28]. Our findings suggest that the SSST can be effectively used to induce subjective stress (and) in adolescent samples as well. The present study was—to our knowledge—also the first to use the SSST paradigm to (successfully) induce stress in sessions with multiple participants simultaneously. We did, however, gauge stress through a subjective and one-item unvalidated measure only. While we were explicitly interested in adolescents’ subjective stress experience in this study, this may have resulted in subjective bias in our findings. Additionally, there are concerns from a developmental perspective about adolescents’ self-awareness to correctly report on their own internalizing mental health status, such as their stress experience (e.g., [29]). Nevertheless, and supporting the construct validity of this one-item measure, participants’ subjective stress increased from T1 to T2, which is in line with the stress induction that was expected through the SSST [27,28]. Moreover, participants’ subjective stress decreased from T2 to T3, which is in line with the stress relief that was expected through physical and/or mental exercise [2,14,15]. Still, future studies using more objective and physiological stress measures are needed to (1) replicate our findings, (2) further investigate the effectiveness of the SSST in inducing stress responses in adolescent samples, and (3) provide more evidence regarding the reliability and (construct) validity of our one-item subjective stress measure.

Some limitations of the present study need to be acknowledged. Data were collected during a science education festival. The experimental setting, therefore, did not mimic a perfect lab environment. Moreover, while the study setting allowed us to collect data from a large sample of secondary school students in a short time frame, our sample may not be fully representative of the broader population of secondary school students. Another limitation of the present study—which is also consequential to collecting data during this science education festival—is that we were not allowed to collect personally identifiable data of participants by our IRB for ethical reasons (i.e., collecting these data in a sample of minors under passive parental consent). As a result, we lack a clear and full demographic description of our sample and cannot indicate to what extent our sample matches the broader population of secondary school students. Moreover, we were therefore unable to correct for potential confounding influences of gender, age, and/or socioeconomic status and to investigate their potential moderating and/or mediating relationships (e.g., their influence on the associations between BIS/BAS and participants’ preferred stress relief strategy). Future studies are needed to investigate these (confounding) relationships in other (potentially more representative) samples. Finally, the reliability of our BAS measure was questionable, and our BIS measure—while acceptable—also did not demonstrate high reliability. Future studies with other BAS and BIS measures are needed to replicate our findings.

## 5. Conclusions

Notwithstanding these limitations, our findings have implications for adolescent stress prevention. Both physical and mental exercise were found to be effective healthy stress relief strategies for adolescents. The effectiveness of these strategies seems unrelated to adolescents’ personalized personality profiles but is influenced by alignment with their personal preferences. As such, the findings of the present study imply that our pre- and intervention efforts for adolescents’ stress-related health problems are better directed at offering them a range of effective and healthy free-choice stress relief activities than on finding them personalized healthy stress relief methods.

## Figures and Tables

**Table 1:** Sample distribution in terms of stress relief activity preference versus performance.

Activity Preference	Activity Performed		
	Mindfulness	Physical Activity	Total
Mindfulness	36	31	67
Physical activity	69	71	140
Total	105	102	207 ^a^

Note. ^a^ Activity preference was not reported by one participant.

**Table 2:** Correlations between all study variables (*N* = 188).

	1.	2.	3.	4.	5.	6.	7.
1. Stress T1	-	**0.45**	**0.27**	**0.36**	−0.02	−0.12	−0.05
2. Stress T2		-	**0.33**	**0.45**	−0.05	−0.05	0.06
3. Stress T3			-	**0.34**	−0.06	**−0.22**	0.05
4. BIS				-	0.05	**−0.16**	0.03
5. BAS					-	0.13	−0.03
6. Activity preference ^a^						-	0.06
7. Activity performed ^a^							-

Note: Bold correlations are significant at *p* < 0.05. ^a^ Activity was coded as 0 = Mental, 1 = Physical.

**Table 3:** Stepwise multivariate linear regression model predicting stress relief (*N* = 188).

	Model 1		Model 2		Model 3A		Model 3B	
	B	95% CI	B	95% CI	B	95% CI	B	95% CI
Constant	**1.31**	**[0.88, 1.75]**	**2.53**	**[0.97, 4.09]**	**2.67**	**[0.52, 4.82]**	**2.60**	**[0.97, 4.23]**
Stress T1	**−0.16**	**[−0.32, 0.00]**	−0.11	[−0.27, 0.04]	−0.12	[−0.28, 0.04]	−0.12	[−0.27, 0.04]
Stress T2	**0.77**	**[0.63, 0.90]**	**0.85**	**[0.70, 0.99]**	**0.85**	**[0.70, 0.99]**	**0.85**	**[0.71, 0.99]**
Activity performed ^a^	−0.21	[−0.84, 0.42]	−0.19	[−0.81, 0.43]	−0.41	[−3.34, 2.52]	**−1.56**	**[−2.61, −0.50]**
BIS			**−0.82**	**[−1.37, −0.27]**	−0.67	[−1.42, 0.08]	**−0.65**	**[−1.19, −0.11]**
BAS			0.24	[−0.26, 0.73]	0.03	[−0.72, 0.77]	0.07	[−0.42, 0.55]
Activity performed ^a^ × BIS					−0.27	[−1.25, 0.71]		
Activity performed ^a^ × BAS					0.38	[−0.62, 1.38]		
Activity preference ^a^							−0.09	[−0.97, 0.78]
Activity performed × preference ^a^							**1.97**	**[0.68, 3.25]**
R^2^	0.43		0.46		0.46		0.50	

Note. Stress relief = subjective stress T2—subjective stress T3. Bold Bs are significant at *p* < 0.05. ^a^ Activity was coded as 0 = Mental exercise, 1 = Physical exercise. CI = Confidence interval.

## Data Availability

Data used in this study will be shared upon reasonable request to the corresponding author and following the data management guidelines.
